# A Few Facts in American Dentistry, Chronologically Arranged

**Published:** 1862-02

**Authors:** J. Richardson


### DENTAL REGISTER OF THE WEST.

Vol. XVI.] FEBRUARY, 1862. [No. 2.

### Original Essays and Communications.

### A FEW FACTS IN AMERICAN DENTISTRY, CHRONOLOGICALLY ARRANGED.

(A paper read before the "Cincinnati Dental Association," February 3d, 1862.)

BY DR. J. RICHARDSON.

I HAVE endeavored in the following pages to group together, in the order of time in which they occurred, some facts in American Dentistry in such manner as shall serve the purpose of ready reference. I am conscious that the list is very incomplete, but I found it impossible to make a fuller statement in the time allotted me. There are, doubtless, many facts scattered throughout the multiplicity of Treatises, Essays, Addresses, Communications and Discussions published in the various books and journals of the country which, in glancing hastily through, I have overlooked, and which I should have been glad to chronicle and credit. The following circumstances have also necessarily very much abridged my statements:

Many facts appear in the history of our profession in this country, which can not be definitely associated with any particular individuals, or to which any precise date can be affixed. There are also many modes of practice, inventions, etc. that could not be intelligibly stated without an extended description of the methods and inventions themselves—an impracticable undertaking in this connection. Such only, therefore, as admit of a brief synopsis have been introduced. Such as appear have been carefully compiled, and may, I think, be relied upon for accuracy.

1766.

Mr. R. Woofendale came from England to New York, and is the first dentist of whom we have any account in the United States. Meeting with but little encouragement in this country, he returned to England in 1768.

1778.

Mr. John Greenwood, the first native American dentist, commenced practice in New York, and is said to have been the only dentist in that city in the year 1790.

1783.

Mr. James Gardette, a surgeon from the French navy, and the first medically educated dentist in the United States, commenced practice in New York, but the following year (1784) went to Philadelphia.

1800.

Dr. H. H. Hayden, first President of the American Society of Dental Surgeons, commenced practice in Baltimore, where he was joined by Dr. Koecker in 1807.

1801.

First dental work published in this country by S. Renners.

1802.

Publication of a popular work entitled "*A Treatise on Dentistry*. By B. T. Longbothom, Surgeon Dentist"

1805.

About this period, Dr. Hudson, of Dublin, commenced the practice of dentistry in Philadelphia.

1819.

Publication of a popular treatise called "*A Practical Guide to the Management of the Teeth, etc.* By L. S. Parmly, Dental Professor."

1821.

Publication of "*Lectures on the Natural History and Management of the Teeth.*" By Dr. L. S. Parmly.

1822.

Publication of "*An Essay on the Disorders and Treatment of the Teeth.* By Eleazer Parmly, Dentist."

1823.

Invention of "*Cheek and Lip Retractor,*" by Dr. J. S. Gunnell.

1824.

Publication of "*A Treatise on the Structure, Diseases and Management of the Human Teeth.*" By Dr. Eleazer Gidney.

1828.

Publication of "*A Physiological Inquiry into the Structure, Organization and Nourishment of the Human Teeth.* By J. Trenor, Dentist."

1829.

Publication of the first systematic treatise in this country, entitled "*A System of Dental Surgery.* By Samuel Shelden Fitch, Dentist."

1832.

Separation of the natural teeth by wedging first practiced by Dr. E. Parmly.

1833.

Publication of a popular treatise entitled "*Remarks on the Importance of the Teeth, etc.* By Francis B. Chewnurg, of Richmond, Va.

1835.

Publication of "*The Family Dentist, or a Familiar Treatise on the Art of Securing a Beautiful Set of Teeth.* By Dr. Homer Bostwick."

1836.

First published account of the use of arsenic for the destruction of exposed dental nerves, by Dr. Spooner, of New York, in a publication entitled "*Guide to Sound Teeth.*" Dr.

Spooner, of Montreal, a brother, was the first to use it for the purpose specified.

Block tin first used as a base for artificial teeth by Dr. W. A. Royce, of Newburgh, N. Y. The manner of using it was subsequently much improved by Dr. Geo. E. Hawes, who introduced the method of casting lower plates from this metal, lately imitated by Dr. Blandy in the "Cheoplastic" process.

Publication of a work called "*Guide to Sound Teeth, etc.* By Dr. Shearjashub Spooner." This work has been spoken of as "the most systematic and elaborate popular treatise which had been published up to the time of its appearance."

1838.

Publication of "*A Public Treatise upon the Preservation of the Teeth, etc.* By M. Overfield."

Publication of "*Dental Hygeia, a Poem on the Health and Preservation of the Teeth.* By Dr. Solyman Brown."

Publication of a treatise entitled "*Observations on the Structure, Physiology, Anatomy and Diseases of the Teeth.* By Drs. Harvy and John Burdell."

1839.

Publication of "*The Dental Art. A Practical Treatise on Dental Surgery.* By Dr. Chapin A. Harris." This is the first edition of the author's well known work called "*Principles and Practice of Dental Surgery,*" and was originally published, before being put into book form, in a weekly literary periodical, published in Baltimore, called the "Monument." It contained 337 octavo pages. The second edition appeared in 1845, under its present title, and was enlarged to some 600 pages. A third edition (750 pages) was issued in 1848—a fourth in 1850—a fifth in 18 —a sixth in 1855—and the seventh and last in 1858, containing 892 pages.

Establishment of the "*American Journal of Dental Science.*" Drs. Chapin A. Harris, of Baltimore, and Eleazer Parmly, of New York, Editors. This journal was published by the American Society of Dental Surgeons down to 1850, when it was transferred to Prof. Harris, who became its sole editor.

First published account of the extirpation of the dental pulp and nerve and fang filling, by Dr. E. Baker. It is affirmed by Dr. Baker, however, that the same operation was performed by Dr. Hudson, of Philadelphia, as early as 1809, but that it was probably confined to the front teeth. The same may be said of Dr. Baker's operations. The first public announcement of this treatment, in connection with the molar teeth, was made by Dr. Dunning in a report on "Practical Dentistry," read before the sixth annual meeting of the American Society in 1845. The operation, however, had been previously practiced by Drs. Maynard, Cherry, Townsend, J. D. White, the Drs. Tucker, of Boston, and others.

1840.

First annual meeting of the "*American Society of Dental Surgeons*," convened in New York, August 18th. Dr. H. H. Hayden, President.

First experiments in bleaching discolored human teeth made by Dr. Dwinnelle. Chloride of soda and lime were the chief agents used.

Publication of a treatise on the "*Preservation of the Teeth, etc.* By David K. Hitchcock."

Letters Patent issued to Alfred Riggs, of New York, for improved Air Chamber for dental plates, described as follows: A plate is struck to fit the mouth, and its central palatal portion perforated with small holes. Over this a plate is struck, forming a chamber about one line in depth at the center, vanishing to the alveolar ridge, and soldered firmly to it. As this plan embodies the essential principle concerned in "Cleveland's Air Chamber," it is not improbable that the latter, which was not patented until some ten years afterwards, was suggested by Dr. Riggs' chamber, the difference consisting simply in the substitution of one large opening in the gum plate (as in Cleveland's) for several small ones, as in Dr. R's.

Establishment of the Baltimore College of Dental Surgery. Faculty: Drs. C. A. Harris, Thos. Bond, jr., H. H. Hayden.

1841.

Publication of "A Treatise on Mechanical Dentistry. By Solyman Brown, M. D.; D. D. S." This work was commenced at the above date in a series of articles contributed to the American Journal, and was the first complete treatise on the mechanical department of dentistry published in this country.

1842.

Organization of the "Virginia Society of Dental Surgeons," Dec. 12th. Incorporated Feb., 1845.

Rimming or banding plates patented by Matt. S. Foster, of Trenton, N. J. The credit of this invention is ascribed by Prof. Harris to Dr. Hullihen, of Wheeling, Va., who practiced it as early as 1836.

1844.

Death of Dr. H. H. Hayden, January 26th, in the 75th year of his age.

First published account of the "Compound screw forceps" for the extraction of roots, contrived by Dr. S. P. Hullihen in 1838. A modification of the same instrument was subsequently (1848) patented by Dr. C. H. Dubbs, of Natchez, Miss.

Publication of "*A Treatise on Diseases of the Mouth.* By J. B. Garrot, M. D." Translated from the French by J. B. Savier, D. D. S.

Translation from the French, by Thomas E. Bond, jr., of "*A New Treatise on the Theory and Practice of Dental Surgery.* By J. Lefoulon, Dentist, Paris."

Publication of "*The Anatomy, Physiology, and Pathology of the Human Teeth, etc.* By Dr. Paul Beck Goddard.

Organization of the "Mississippi Valley Association of Dental Surgeons."

1845.

Publication of "*A Popular Treatise on the Teeth.* By Robert Arthur, D. D. S."

Translation from the French, by Prof. Chapin A. Harris, of "*A Treatise on Second Dentition, etc.* By C. F. Delabarre."

The operation termed "*Risodontrypy*" first performed by S. P. Hullihen, of Wheeling, Va.

Translation from the French, by Dr. Robert Arthur, of "*Anatomy of the Dental System, Human and Comparative.* By P. F. H. Blandin."

Establishment of the "Ohio College of Dental Surgery." Faculty: Drs. James Taylor, Jesse W. Cook, Melancthon Rogers.

Adoption of the famous *amalgam* resolutions by the American Society of Dental Surgeons, declaring the use of any amalgam for filling teeth malpractice, and pledging each member not to use them under any circumstances, under penalty of expulsion from the Society. The same resolutions were rescinded by the Society at its eleventh annual meeting in 1850.

Valvular air chamber, applied to suction plates, contrived by Dr. W. H. Dwinnelle.

1846.

Organization of the "Pennsylvania Association of Surgeon Dentists," Dec. 15th. Dr. G. A. Planton, President.

Dental Ring and Drill Socket, consisting of a ring to be worn on the fore or middle finger, to which is attached a cup or socket, to receive the upper end of the drill, and to prevent it from hurting the hand—invented by Dr. A. Westcott, of Syracuse, N. Y.

First successful experiment with sulphuric ether as an an-

æsthetic in extracting teeth, by Dr. W. T. G. Morton, dentist, Boston, Mass. Chloroform was first used as a substitute for ether in producing insensibility by inhalation, by Prof. J. Y. Simpson, of Edinburgh, in the fall of 1847.

"Cleveland's Air Chamber," applied to suction plates, contrived by Dr. J. A. Cleveland, of Charleston, S. C. Patented in 1850.

Publication of first American from third London edition of Fox's "*Natural History and Diseases of the Human Teeth.*" Edited by Prof. Chapin A. Harris.

Publication of the first number of the "*New York Dental Journal,*" a monthly, edited by Dr. C. C. Allen.

1847.

Translation from the French, by Prof. C. A. Harris, of "*Complete Elements of the Science and Art of the Dentist.* By M. Desirabode."

Publication of "*A Practical Treatise on the Operations of Surgical and Mechanical Dentistry.* By Dr. Samuel C. Herbert."

Publication of a treatise entitled "*Teeth—their Structure, Diseases, and Treatment.* By Dr. John Burdell."

Publication of the first number of the "*Dental Register of the West,*" a quarterly, published by the Mississippi Valley Association, and edited by Dr. James Taylor, of Cincinnati, and Dr. B. B. Brown, of St. Louis, Mo.

Publication of the first number of the "*Dental News Letter,*" a quarterly journal, published by Messrs. Jones, White & Co., Philadelphia, Pa.

Organization of the "*South New Jersey Dental Association,*" July.

The earliest period mentioned by Dr. Atkinson at which he employed the *mallet* for condensing gold in filling teeth. He ascribes its *first* use to Dr. E. Merrit, of Pittsburgh, Pennsylvania.

1848.

Gutta percha first recommended as a material for taking impressions of the mouth by Dr. F. J. Colburn, of Newark, N. J., and Dr. W. P. Blake, of New York City.

Publication of "*A Popular Treatise on the Teeth, etc.* By Mayo G. Smith."

Publication of "*The Principles and Practice of Dentistry Simplified and Condensed, etc.* By R. D. Addington, D.D.S."

"*Gilbert's Central Cavity Plate*" patented in February by Levi Gilbert, of New Haven, Conn.

1849.

Publication of "*A Dictionary of Dental Science, Biography, Bibliography and Medical Terminology.* By Professor Chapin A. Harris."

"*Lateral Cavities,*" applied to atmospheric pressure plates, recommended by Dr. J. F. B. Flagg.

"*Hawes' Moulding Flask*" contrived by Dr. G. E. Hawes, of New York.

First annual meeting of the "*Society of the Alumni of the Baltimore College of Dental Surgery,*" held in Baltimore, March 1st. Dr. H. Colburn, President.

Translation from the French, by Dr. Philip H. Austen, of "*A Treatise on the Diseases and Surgical Operations of the Mouth and Parts Adjacent, etc.* By M. Jourdain."

"*Hill's Soft Stopping,*" patented Feb. 13th.

A method of encasing loose teeth in plaster of Paris preparatory to plugging or filing first suggested by Dr. C. C. Allen, Editor of *Dental Recorder.* The same method was subsequently *re-discovered* by Dr. W. A. Helm.

1850.

Death of Dr. John Burdell, March 11th, aged 44 years.

The use of gum elastic rings in regulating the teeth first recommended by Dr. E. G. Tucker, of Boston, Mass.

First experiments in the use of "*sponge gold*" for filling carious teeth.

"*Transylvania School of Dental Surgery*" at Lexington, chartered by the Kentucky Legislature. Faculty: Drs. J. B. Stout, John B. Lindsay, B. J. Dudley, Jas. S. Drane, G. W. Evans.

1851.

First published description of the method of electro-plating, or depositing plates for mounting artificial teeth by the electrotype process, by Dr. Arthur Hill.

Publication of "*A Practical Treatise on Dental Medicine.* By Thos. E. Bond, A. M., M. D."

Publication of a treatise on "*Ether and Chloroform.* By Dr. J. F. B. Flagg."

"*Philadelphia College of Dental Surgery*" chartered, and the following Faculty elected, October 17th: Drs. J. D. White, Ely Parry, Elisha Townsend, Robert Arthur, J. L. Buckingham.

"*New York College of Dental Surgery*" instituted with the following Faculty: Drs. A. Westcott, A. B. Shipman, Thos. Spencer, R. F. Stephens, Daniel Van Denburgh.

Flax cotton first recommended for drying cavities preparatory to filling by Dr. Alphonso Severence, of Great Falls, New Hampshire.

Specimens of American dental mechanism exhibited at the great Industrial Fair in London, England, by Drs. Allen, Hunter, Reynolds, Buckingham, Barlow, Hitchcock, and others; and by Messrs. Jones, White & M'Curdy, Alcock, Stockton, and Chevalier.

Letters Patent issued to Dr. John Allen for a "new mode of setting artificial teeth on metallic plates," (known as "continuous gum work") in December.

Description of a new mode of filling cavities of difficult access in the posterior proximate surfaces of the second and third molar teeth, by Dr. F. H. Badger, of Nashville, Tenn. A cylinder of gold foil is made just large enough to enter readily the cavity of decay,—this is then introduced, and its center pierced forcibly with a sharp-pointed instrument, then using successively those with larger points until the gold is forced into close contact with the walls of the cavity. The central opening is then filled with a pellet of gold of the required size, and the whole condensed and finished in the usual way.

1852.

Formation of the "*Ohio Dental College Association*," February 19.

Organization of the "Allegheny County (Pa.) Dental Association" at Pittsburgh, October 16th. W. M. Wright, President.

1853.

Publication of "*A Text Book of Anatomy and Guide in Dissections, for the Use of Students of Medicine and Dental Surgery*. By Prof. W. R. Handy, M. D."

Publication of a work on "*Dental Chemistry and Metallurgy*. By A. Snowden Piggot, M. D."

Death of Josiah Foster Flagg, Dec. 20th.

Letters patent issued to Dr. Alfred J. Watts, of Utica, N. Y., for a "*new and improved mode of preparing and crystallizing gold*," April 26th. It was in this or the preceding year that a practical article of sponge or crystal gold for filling teeth was first obtained—in England by Mr. Joseph Barling, and in this country by Dr. A. J. Watts. It is affirmed by Dr. Dwinelle that Dr. C. F. Jackson, of Boston, took the initiative in the production of this form of gold, but that the article obtained by him was not an available material for the uses of our profession—that the English gold of B rling, though structural, was not crystal-formed, and that Dr. A. J. Watts' gold alone was crystalline. A very well approved article of crystalline gold was also manufactured by Drs. Watt and Taft soon after the first appearance of this article in the market.

Publication of a work entitled "*Treatment of Dental Caries, Complicated with Diseases of the Pulp.* By R. Arthur, M. D., D. D. S."

1854.

Letters patent issued to Dr. Mahlon Loomis, Cambridgeport, Mass., for a process of constructing artificial dentures exclusively of porcelain.

"*Saliva Pump*," patented by F. Davison, Jan. 17th. The design of this instrument is ascribed to Prof. R. Arthur.

Formation of the "*Vermont Society of Dental Surgeons*" at Montpelier, Oct. 25th.

1855.

*Perchloride of gold* recommended as an application to sensitive dentine, by Profs. Taft and Watt.

Celebrated trial in the United States Circuit Court for the Southern District of Ohio, in the case of Dr. John Allen *vs.* Dr. W. M. Hunter, for alleged infringement of patent. Two points were involved, the validity of the patent, and the infringement by the defendant. The patent was sustained, but the point of infringement was not.

First meeting of the "*American Dental Convention*," held in the city of Philadelphia, Aug. 2d. Dr. J. B. Rich, of New York City, President.

Gutta percha proposed as a base for artificial teeth, by Dr. N. B. Slayton, of Madison, Indiana.

Dr. J. B. Branch's apparatus for producing local anæsthesia by congelation first introduced to the notice of the profession.

Prize of $100 awarded by the Mississippi Valley Association to Dr. Geo. Watt, of Xenia, for the best "*Essay on Dental Surgery, adapted to popular circulation.*"

New mode of using gold foil in filling teeth, recommended by Prof. Arthur, viz: Roll up foil very loosely into rope form; pass it quickly through the flame of a spirit lamp; cut it up into small pieces, and pack it into the cavity to be filled, with sharply serrated instruments, condensing each piece as it is put into place.

1856.

Formation of the "*Western Dental Society*" in April.

Commencement of the "*Dental Obturator*," a quarterly journal, edited by Dr. John S. Clark, New Orleans, La.

Publication of the "*Forcep*," a dental periodical, New York.

Sixteenth Annual Meeting and Dissolution of the American Society of Dental Surgeons at New York, Aug. 7th.

Organization of the "*St. Louis Dental Society*," December 16th.

Organization of the "*North Carolina Dental Society*" at Raleigh, Oct. 17th.

Dr. Putnam says, "the first apparatus (for vulcanizing) which is now in use for dental purposes, was established in May," and "that as to *priority* in its application to artificial dentistry, *that* is wholly creditable to Mr. Charles Goodyear, jr., and Mr. F. A. Bevin, neither of whom are dentists."

Process of mounting artificial teeth known as "Cheoplastic" introduced to the notice of the profession by Dr. A. A. Blandy.

1857.

Dr. Slayton's method of plating silver, copper or brass with gold foil alloyed with quicksilver communicated to the profession.

Publication of "*A Treatise on the Use of Adhesive Gold Foil.* By Dr. Robert Arthur."

Death of Dr. C. C. Allen, May 30th, for many years editor of the "Dental Recorder."

Death of Dr. Harvy Burdell.

Death of Dr. S. P. Hullihen, of Wheeling, Va., March 27.

The attention of the profession first directed to the use of the *tincture of iodine* in chronic periodontitis, accompanied with constant purulent discharge, by Dr. Garretson, of Philadelphia. It is affirmed, however, to have been employed by Dr. Atkinson, of Cleveland, for the same purpose as early as 1854, and Prof. George Watt claims to have used it at a still earlier period.

1858.

Galvanism first recommended and applied in the extraction of teeth, to induce insensibility to pain.

Establishment of the "*New York Dental Journal.*" Edited by Frank H. Norton and Geo. H. Perine.

Formation of the "*Indiana State Dental Convention*" at Indianapolis. Dr. J. F. Johnson, President.

Death of Dr. Elisha Townsend, Oct. 13th, aged 54 years.

The treatment of recently exposed dental nerves, preparatory to filling, with nitric acid recommended by Dr. Wright, of Pittsburgh, Pa.

1859.

Experiments first made in casting aluminum plates as a base for artificial teeth.

Commencement of the publication of the "Dental Cosmos" (successor to the "Dental News Letter"), a monthly periodical, edited by Drs. J. D. White, J. H. McQuillen and Geo. J. Zeigler.

Published account of Prof. Geo. Watt's method of filling a tooth complicated with a dead pulp or nerve—briefly stated thus: Having cleansed the canal of the root as perfectly as possible of all organic matters, a pledget of cotton moistened with creosote is introduced into the canal and confined temporarily with dry cotton for a day or two. The same application is then renewed and secured in the cavity with wax for one or two days, when it is removed, the canal thoroughly washed out with water,—afterwards with chloroform and dried. The cavity of decay is then filled, leaving the fang or fangs unfilled.

Organization of the "Georgia Dental Society."

Organization of the "American Dental Association" at Niagara Falls, Aug. 3d.

Invention of an instrument called a "*Micrometer*," for testing the expansion of plaster of Paris, by Professor T. L. Buckingham.

Formation of the "New York State Dental Association," Sept. 6.

Publication of "A Practical Treatise on Operative Dentistry." By Prof. J. Taft.

Introduction of *osteoplastic* preparations for filling teeth.

Formation of the "Mad River Valley Dental Association," November, at Springfield, Ohio.

"Dental Register of the West," a quarterly publication, converted into a monthly periodical at the close of the 12th volume. Edited by Profs. J. Taft and Geo. Watt.

Introduction of Dr. Slayton's method of uniting porcelain teeth to a plate, and forming a rim to the same by means of an amalgam of gold or silver.

Vulcanized Rubber first employed by Dr. G. C. Hunt, of Indianapolis, Ind., to unite artificial teeth to gold base, thus dispensing with use of solder for that purpose. The process was subsequently patented by Drs. A. M. and J. J. Asay, of Philadelphia.

1860.

Publication of the "Dental Instructor," a quarterly periodical, edited by E. A. L. Roberts, of New York.

Publication of the "Vulcanite," a quarterly journal, edited by Dr. B. W. Franklin, New York.

Formation of the "Northern Ohio Dental Association" at Cleveland, March 6th.

Formation of the "Kentucky State Dental Association," at Lexington, April 24.

Publication of the "Southern Dental Examiner." Edited by Dr. J. P. H. Brown, Atlanta, Ga.

Death of Dr. Alvin Blakesly, of Utica, N. Y., on his way to Savannah.

Introduction into dental practice of perchloride of iron as a hæmostatic,—more recently, the persulphate.

Death of Prof. Chapin A. Harris, of Baltimore, Sept. 29.

Publication of "A Practical Treatise on Mechanical Dentistry. By Prof. J. Richardson."

*Paraffine* recommended as a material for taking impressions of the mouth, by Dr. R. Peckover, Paris, Ky.

1861.

Organization of the "New Orleans Dental College: Faculty: Drs. John S. Clark, James S. Knapp, Geo. J. Friedricks, N. B. Benedict, Sanford S. Riddle, A. F. McLain, W. S. Chandler.

A "*Retaining Instrument*," designed to hold in place the first piece or pieces of gold introduced into a cavity in filling, contrived by Prof. J. H. McQuillen.

Formation of the "Central Ohio Dental Association" at Columbus, Jan. 15th.

The following facts, not susceptible of strict chronological arrangement, are appended, as having some historical interest.

Near the beginning of the present century, Dr. Jas. Gardette, dentist, of Philadelphia, substituted elastic flat gold bands (clasps) in the place of ligatures of silk or fine gold wire, at that time generally used to secure partial sets of artificial teeth in the mouth. It is also claimed by his biographer, Emile Gardette, that he was the first to avail himself of the principle of atmospheric pressure for the retention of entire sets of teeth, and is said to have successfully applied this principle for the purpose specified as early as 1800. Dr. G. returned to his native village of Agen, in France, in 1829, and subsequently taking up his residence in Bordeaux, died in August, 1831, at the advanced age of seventy-five.

Prof. Harris, so far as I can ascertain, was the first to recommend the use of chloride of zinc to obtund the sensibility of dentine, in 1853; and to Prof. Arthur is due the credit of having suggested the substitution of cobalt (crude arsenic) for the arsenious acid for the same purpose.

*Asbestos*, as a non-conductor of caloric in filling teeth, was first brought to the notice of the profession by Dr. Solyman Brown, in a paper published in the American Journal in 1840. Experiments were made with gutta percha for the same purpose by Prof. Harris some time prior to 1840, and the results of his experience communicated to the American Society in 1848. Horn, pared or filed, was recommended for similar purposes in 1854, by Dr. C. A. Du Bouchet.

Dr. Clark, of New Orleans, in a communication to the "Dental Register," in 1852, describing his method of crown and fang filling with cylinders of gold foil, gives the credit of originality in this method to Dr. Badger, of Nashville, Tenn., but gives no intimation of the time when the latter first practiced it. Dr. James Taylor, two years previous to the publication of Dr. Clark, in an address before the Mississippi Valley Association on filling teeth, describes a method of filling with blocks or cylinders, in all essential respects the same as that practiced by Dr Clark, and was the first published account of the method.

*Corundum* wheels were first manufactured in this country by Messrs Jones, White & Co., of Philadelphia.

Publication of "Practical Hints on the Teeth." By W. E. Ide, of Columbus, Ohio. A Popular Treatise, the date of which I am unable to give.

In 1830, there were probably not more than 300 dental practitioners in the Union. In 1842 the number had increased to about 1,400. In 1848, about 2,000, and at this period of time there are probably not less than 6,000.

A statement appeared in the News Letter of 1853 that "at least a million and a half of porcelain teeth are manufactured annually in this country."

Dr. A. A. Planton, who located in Philadelphia in 1817, is said to have been the first to introduce mineral teeth into the United States,—the date of their introduction being somewhere between 1817 and 1819.

The credit of having first *manufactured* mineral teeth in this country rests between the elder Mr. Peale and Dr. Planton, as they were both experimenting in this department at the same time, 1819. Dr. Joseph E. McIlhenny, of Philadelphia, in answer to a communication addressed to him by Dr. McCurdy, makes the claim of priority, but by his own showing did not commence his experiments until 1826.

The first use and recommendation of plaster of Paris for taking impressions of the mouth is ascribed by Dr. Harris to Drs. Westcott and Dunning.

